# Carbon Pricing is not Unjust

**DOI:** 10.1002/gch2.202300089

**Published:** 2023-12-10

**Authors:** Kian Mintz‐Woo

**Affiliations:** ^1^ Department of Philosophy & Environmental Research Institute University College Cork Cork T12 Y1F1 Ireland; ^2^ Equity and Justice Group International Institute for Applied Systems Analysis Laxenburg A‐2361 Austria

**Keywords:** carbon pricing, carbon taxes, climate ethics, climate justice, distributive justice

## Abstract

The aim of this perspective is to argue that carbon pricing is not unjust. Two important dimensions of justice are distributive and procedural (sometimes called “participatory”) justice. In terms of distributive justice, it is argued that carbon pricing can be made distributionally just through revenue recycling and that it should be expected that even neutral reductions in emissions will generate progressive benefits, both internationally and regionally. In terms of procedural justice, it is argued that carbon pricing is in principle compatible with any procedure; however, there is also a particular morally justifiable procedure, the Citizens’ Assembly, which has been implemented in Ireland on this precise question and has generated broad agreement on carbon pricing. It is suggested that this morally matters because such groups are like “ideal advisors” that offer morally important advice. Finally, an independent objection is offered to some ambitious alternatives to carbon pricing like Green New Deal‐type frameworks, frameworks that aim to simultaneously tackle multiple social challenges. The objection is that these will take too long to work in a climate context, both to develop and to iterate.

## Graphical abstract

Some have suggested that policies for pricing carbon are unjust. But what does “unjust” mean? This Perspective argues that carbon pricing polices are not unjust, both distributionally (whether carbon pricing is net regressive) and procedurally (whether carbon pricing would be endorsed by ideal informed observers). Finally, it defends carbon pricing policies from the objection that they are insufficiently systemic changes.

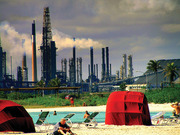

## 1 Introduction

A major source of tension in society about climate policy revolves around whether to support or adopt climate pricing policies. Climate pricing includes both carbon taxes (“pricing instruments”), where a fixed cost is added for each ton of carbon dioxide emitted, and cap‐and‐trade systems (“quantity instruments”), where a fixed volume of emissions (a “cap”) is subjected to allowances that can be bought and sold (“traded”). Since the volume is fixed, cap‐and‐trade systems also generate a carbon price, albeit indirectly.

In ideal contexts, pricing and quantity instruments perform the same function. Furthermore, in ideal contexts, they apply uniformly and cannot be avoided (e.g., through exemptions). Why is this ideal? There is a political and a philosophical answer. The political answer is that a uniform carbon price avoids the optics of a government picking certain industries or firms and privileging them over others. The philosophical answer is that it would be unfair: a carbon price is meant to disincentivize carbon emissions. If some embedded emissions are priced less highly than others, then this would incentivize more emissions in those contexts and, furthermore, would not give a market signal to find less carbon‐intensive production methods, *wherever* they may be found. Furthermore, the theory of Pigouvian taxes requires that fungible quantities are taxed uniformly; divergent carbon prices would suggest that the same pollutant generates different negative externalities, even though CO_2_ mixes uniformly in the atmosphere. If the price in a pricing instrument is optimal, it will lead to an optimal volume of emissions (i.e., emissions such that the social benefit exceeds the social (climate) cost). If the cap in a quantity instrument is optimal, it will lead to an optimal price on allowances, since firms will be willing to pay for allowances up to the point at which it is no longer profitable to emit. If designed carefully, both involve payments to the government (carbon taxes directly and cap‐and‐trade systems if the allowances are auctioned off), and the resultant government revenue can be used for a range of purposes. Although we are not in ideal contexts, and there are differences between these instruments in practice, their differences are less important with respect to the issues of justice I am interested in here. (Aside from these issues of justice, there are some other morally important differences between these types of carbon prices—as well as differences with regulatory command‐and‐control instruments—which are beyond the scope of this perspective. I canvass a variety of them elsewhere^[^ ^19^ ^]^ and some other moral justifications for carbon pricing are based on the right to energy and the duty not to harm.^[^ ^53^ ^]^)

In short, I will focus on a moral issue that has received a large amount of attention: is carbon pricing unjust? Some campaigners and civil society groups, especially those involved in environmental and climate justice spaces, have rejected carbon pricing as unjust. This claim deserves some discussion and, in this perspective, I discuss a few potential dimensions of justice that could be relevant to this claim. My goal is to show that, for the most important dimensions of justice, carbon pricing is not unjust as a policy instrument for addressing climate change. This is not to say that carbon pricing addresses climate change alone—it is best thought of as part of a portfolio of climate policies that could be supplemented with things like behavioral nudges^[^ ^1^ ^]^ and investments in climate innovations.^[^ ^2^ ^]^

I focus on two forms or dimensions of justice: distributive justice and procedural justice (sometimes called “participatory justice”). Distributive justice has to do with what we owe to each other in contexts with limited goods or resources. Procedural justice has to do with how decisions are made and whether they are representative, especially of particularly vulnerable groups. For instance, procedural justice can involve discussions of democratic legitimacy. There are other dimensions of justice, but those others are much more contested—and many of them are also less relevant or determinate in the sense that it is less clear how they apply in this context or how distinctive the issues they raise are. (For instance, political discussions often employ the term “social justice”, but social justice includes both distributive justice—in terms of attending to and lessening socio‐economic vulnerability—and procedural justice—in terms of making sure that civil society and ordinary people are able to affect the policies and decisions that they may have a stake in. In my view, the term “social justice” does not identify a new form of justice but implicitly combines a variety of justice dimensions, thus making it more difficult to focus on any of them.)

## 2 Dimensions of Justice and Carbon Pricing

Before considering the justice of carbon pricing, it is worth trying to understand some of what the campaigners objecting to carbon pricing are saying. To illustrate, we can begin with the Green New Deal, which has attracted a lot of support among campaigners in the United States (and can be contrasted with policies like carbon pricing). While not a policy proposal (or even a set of policy proposals), the Green New Deal (as discussed in United States House Resolution 109) sets forth an ambitious policy *framework* or set of goals, including guaranteed wages, high‐quality healthcare, adequate housing and access to a clean environment.^[^ ^3^ ^]^ In a book arguing for a Green New Deal‐type framework, Naomi Klein writes that:

given how far down [the road to climate crisis] we are, there is no point pretending that [options] are going to be easy. It's going to take a lot more than a carbon tax or cap‐and‐trade. It's going to take an all‐out war on pollution and poverty and racism and colonialism and despair all at the same time.[4, pp. 50–51]

This type of cri du coeur exemplifies the goals of some critics of carbon pricing; they prefer to support framework proposals that ambitiously tackle a variety of social ills that they believe are inextricable from climate harm. (However, even here, it is important to note that there may be more agreement than is apparent; for instance, contra Klein, proponents of carbon pricing do not think that it will only take carbon pricing policies to address climate change^[^ ^1^ ^]^ so Klein might be objecting to a strawman opponent.)

Since, like all other policies, carbon pricing will not adequately address climate change in isolation, the question is would carbon pricing increase the likelihood of just outcomes in terms of various forms of justice, whether distributive and procedural (or racial and gender and recognitional and…)? More precisely, would carbon pricing increase this likelihood compared to the status quo or compared to a robust, Green New Deal‐style, policy framework?

It is true that, if we endorse the goals of the Green New Deal‐style framework and if it were to be successfully adopted and enacted, it would have much greater direct effects on society and address far more forms of injustice. However, there are two caveats. In this section, I offer a first caveat: that carbon pricing can also, albeit indirectly, promote several dimensions of injustice, such as distributive and racial justice, potentially laying the groundwork for greater gains, so the comparative advantage of Green New Deal‐style frameworks is not as great as they may initially appear (§2.1). Furthermore, I will defend the claim that carbon pricing is consistent with procedural justice—indeed, we have some reason to believe that procedurally just, democratic processes would positively *support* carbon pricing policies (§2.2). In the following section, I offer a second caveat: that the evaluation of policy options should reflect their fit to the problems they are meant to solve (§3). I argue that the timescale of large‐scale social change is a poor fit to address the climate challenge.

### 2.1 Distributive Justice

It is certainly the case that fairness and distributive justice matter for the acceptance of carbon pricing policies.^[^ ^5^, ^6^ ^]^ If so, it matters whether carbon pricing can promote distributive justice. I will argue that it can if revenue recycling is used to offset the regressivity of the initial incidence of carbon prices. Many intuitively believe that the initial incidence of carbon prices is unfair or will be regressive, i.e., will disproportionately fall on those with lower incomes,^[^ ^7^ ^]^ but this intuition fails to take into account the capacity to redistribute revenues from carbon pricing.^[^ ^8^ ^]^

We can start with the initial incidence of carbon prices, meaning the effect of the carbon prices alone and how regressive or progressive (whether this effect is borne disproportionately by the less or more well‐off). That the *initial* incidence is regressive is broadly true in *developed* economies—but is not internationally true (e.g., in countries where only the wealthy can afford private cars, increases in oil prices will affect the wealthy but miss affecting those less well‐off, generating an overall progressive effect).^[^ ^9^, ^10^ ^]^

Whether a policy is overall regressive or progressive is agreed to be of importance across the spectrum of distributive justice theorists. In short, this is because most justice theorists believe that current levels of inequality are unjust. Furthermore, distributive justice theorists, ranging from utilitarian and prioritarian to sufficientarian and egalitarian, endorse the importance of reducing material inequalities, albeit for different reasons.^[^ ^11^, ^12^ ^]^ Utilitarians believe that redistribution from the wealthiest to the poorest would more effectively generate utility or welfare.^[^ ^13^ ^]^ Prioritarians agree but believe, moreover, that we should weight the utility or welfare of those with less even more strongly than the welfare benefits of redistribution imply—justifying even greater redistribution. Sufficientarians are concerned about those with low levels of resources who fall below some basic or minimal threshold, which justifies redistribution toward those in poverty since poverty on most accounts brings people below that basic or minimal threshold.^[^ ^14^ ^]^ Finally, egalitarians believe that it is intrinsically valuable for resources to be equally distributed. For all of these theorists, therefore, distributive justice would be served by redistribution from those with the most to those with the least. In short, then, a policy is distributively *unjust* if the net effect is to increase inequality (i.e., if the policy has regressive effects), while a policy is distributively just if the net effect is not to increase inequality (i.e., if the policy has proportional or progressive effects).

However, when we turn to the overall effect of carbon pricing, including both the initial incidence and the effects of revenue recycling, it may be progressive or *increasing* of distributive justice, since the revenues can be distributed to offset any regressivity from the initial incidence. For instance, Budolfson et al.^[^ ^15^ ^]^ find that aggressive mitigation spurred by a high carbon tax could lead to meeting the 2 °C target goal—while reducing global inequality and global poverty by employing a basic equal per capita rebate. In other words, even if the initial incidence of a carbon price is negative—and the revenue recycling is not intentionally progressive (equal per capita distributions are not responsive to current heterogeneity)—the net impact can be significantly progressive (this work reinforces messages from others^[^ ^16^ ^]^).

It is worth mentioning an often misunderstood point. Some campaigners^[^ ^17^ ^]^ point to massive estimates in carbon subsidies (e.g., the IMF found, for 2021, an estimated global USD $5.9 trillion in fossil fuel subsidies) and ask why we need a carbon tax when “there is more than enough money lying around in the public purse”. The issue is that, in everyday language, a “subsidy” is when the government actively pays to reduce costs for producers or consumers. However, estimates like the IMF's comprise both explicit and implicit subsidies.^[^ ^18^ ^]^ Explicit subsidies are ones that are the active payments we might expect, but implicit subsidies are simply passive *failures* to properly price. Not only is this so, but the implicit subsidies also form the vast bulk of these estimates (e.g., in the IMF case, the explicit subsidies are ≈8% of the total, and implicit subsidies are ≈92%). “Removing” implicit subsidies just *is* carbon pricing—and it is a significant commitment to carbon pricing! In short, although not everyone understands this, it is logically inconsistent to be against removing (explicit and implicit) subsidies and to be against carbon pricing.

It is also important to keep in mind that this progressive impact is a morally important *indirect* effect of carbon pricing; the primary goal of carbon pricing policy is to disincentivize emissions that are not sufficiently valuable. The idea here is that carbon pricing is meant to incentivize optimal pollution. This may sound like a contradiction in terms—how could it be optimal to pollute the environment?—but it is clear that in some circumstances it is socially valuable to produce emissions. For instance, if a person needs to get to a hospital quickly, emissions associated with driving that person are (in most if not all cases) socially justifiable. Why is this? It is because the social benefits of saving that person are much greater than the (real!) costs of increasing climate change. By making individuals pay the costs of climate change, they will get closer to making socially optimal decisions, i.e., ones where the social benefits (of their socially or financially productive activities) outweigh the climate harms that their emissions contribute. In many cases involving carbon generation today, people are not producing socially valuable outcomes on net; if an appropriate carbon price is introduced, many of these people will change their behavior. These shifts in behavior are likely to get us closer to optimality or efficient outcomes.

But even given that changing incentives is merely an indirect effect, it is notable that carbon pricing could promote distributive justice if the revenues are recycled properly. This point is fairly abstract, so it is worth demonstrating with a simple numerical example:

Suppose you are much richer than I am and spend $10000/month. I only spend $1000/month. A carbon tax is introduced and, because the initial incidence of a carbon tax is regressive, it hits me harder. Let us say that you end up being taxed effectively at 5% so you spend $500/month on this carbon tax. However, we assumed that the initial incidence is regressive since, for instance, more of my monthly spending is on products like gas. Suppose I end up being taxed 10% or $100/month. Now let us suppose the government simply divides up all the revenue and, using equal per capita distribution (i.e., without reference to anyone's wealth or contribution size), provides both of us with [$500/month + $100/month]/ 2 people = $300/month/person. You end up net $200 down (−$500 + $300/month) but I end up $200 up (−$100 + $300/month). In other words, even though the initial incidence of the tax we assumed to be regressive and even though we rebated the tax revenue in a non‐progressive (simply flat) way, the net result is still a progressive transfer from the richer to the poorer!^[^ ^19^ ^]^

One could respond by saying that there can be many other distributional effects beyond the tax incidence of a carbon price, ranging from sectoral impacts for carbon‐intensive industries to consumer impacts for different purchasing behaviors. So is it unfair or unjust that workers in carbon‐intensive (brown) sectors would have job impacts while workers in sustainable (green) sectors would benefit from greater demand?

While I certainly acknowledge these differential effects, I do not think they raise special questions of justice. The reason that I do not think these effects are morally unjust is straightforward; they are the result of the (implicit) social subsidies that were not warranted previously. These subsidies made brown production and consumption behavior more inexpensive than their social cost.

A critic could continue this line of questioning about whether this is unfair by suggesting that it is a shock to workers who trained and legitimately expected that their industries would continue to exist,^[^ ^20^ ^]^ but I do not believe that workers are entitled to their jobs continuing. Sometimes demand shifts, and shifting demand due to social value is equally, if not more, justified as shifting demand due to consumer preferences.

Indeed, one could take a stronger position. Both philosophers^[^ ^21^ ^]^ and economists^[^ ^22^ ^]^ have argued that pricing policies should not be neutral between consumption behaviors, but instead provide extra disincentives to high carbon lifestyles over and above a good‐insensitive carbon price.^[^ ^23^ ^]^

Note that Budolfson et al.’s modeling suggests that carbon pricing policies are also consistent with meeting an ambitious target, like the 2°C target.^[^ ^15^ ^]^ This addresses the concern that some campaigners have, one which Boyce^[^ ^24^ ^]^ calls the “false solution” objection: that carbon prices cannot make a meaningful difference in emissions. Although the primary purpose of this paper is to address justice considerations, it is worth pointing to some of the relevant empirical literature. On the one hand, there are concerns about how much carbon pricing has affected behavior empirically,^[^ ^47^ ^]^ although the data are noisy and current carbon prices are quite low. Overall, the literature suggests that the existing (overwhelmingly low) carbon prices have had significant, albeit modest, effects thus far.^[^ ^48^, ^49^, ^54^ ^]^ On the other hand, in favor of carbon pricing, some particular cases, such as the carbon tax in British Columbia, suggest that a well‐designed carbon tax can be broad‐based and reduce emissions relative to pre‐tax pathways^[^ ^50^ ^]^ without loss of jobs.^[^ ^51^ ^]^ In this case, at least, there is an impressive template for success.

Not only are carbon prices compatible with promoting distributive justice, but they may also be useful in promoting racial justice. The reason is straightforward: climatic impacts are likely to disproportionately affect vulnerable groups (e.g., racial minorities); more relevant in this discussion, if it was not the case, then climate change would not introduce any special racial justice challenges. This is true globally, where the Global South faces disproportionate climate harms compared to the Global North, and also regionally, where vulnerable groups tend to be more exposed and less able to adapt.^[^ ^25^ ^]^ Supposing that individuals in an economy respond to economic incentives, carbon pricing would reduce emissions—and, ultimately, climatic impacts. Policies that address climate impacts in neither intentionally progressive nor regressive ways could be expected to have roughly proportional benefits to vulnerable groups (at least in climate terms since greenhouse gases are well‐mixed in the atmosphere). Since the burdens of climate harm are regressive, proportional benefits would be progressive.

This argument applies to co‐harms, like local co‐pollutants as well—indeed, probably even more strongly. Since vulnerable groups (especially racial minorities in the United States) are disproportionately exposed to local pollution (e.g., PM2.5 and NOx) from pollution point sources like factories and power plants,^[^ ^26^, ^27^, ^28^ ^]^ reduction in emissions activities that reduce these co‐pollutants would have even more than proportional benefits to these groups (for simplicity, we can say that there would be progressive co‐benefits). Even if the reduction of co‐pollutants is regressive, it would *still* have progressive co‐benefits as long as the regressivity of the reductions is less than the regressivity of the initial disproportionate exposure. (While I am not aware of research that tries to determine how regressive and progressive these effects are—or what their net effect is—this would be a valuable route for future work.)

Some have argued that this is overly optimistic and that we should expect that reductions in emissions activities would not have neutral effects, but highly regressive effects. Boyce et al., for instance, point to research that suggests that, under cap‐and‐trade systems in California, some of the facilities that generated the largest increases in emissions had high proportions of racial minority groups in their vicinity.^[^ ^16^ ^]^ However, as with most instances, there were a variety of different regulations (as Boyce et al. admit), so disentangling the impacts of one policy is challenging, especially given the limited number of cases and the fact that considering the facilities with the greatest increases may give a distorted view of the overall effects. More broadly, the short‐term effects are likely to be noisier than the long‐term trend, especially if carbon prices increase over time (as they are expected to do in California). Regardless, Boyce et al. are certainly correct that this justifies monitoring air quality near racial minority groups in order to determine how regressive or progressive these effects are, even if it is somewhat soon to draw conclusions about the impacts of pricing policies on co‐pollutants.

I conclude that there is a strong case to make that carbon pricing would not set back distributive justice in terms of disproportionate impacts on socioeconomic and vulnerable groups, for instance, in terms of race. The main reason is that climate change can be expected to threaten (independently) vulnerable groups more seriously, so even proportional reductions would be progressive.

### 2.2 Procedural Justice

When considering procedural justice (or “participatory justice” as it is sometimes called), the case is even more straightforward that carbon pricing is not unjust. The reason is that “carbon pricing” is simply the name for a set of policy instruments, not a way of *choosing* policy instruments. In other words, these instruments are compatible with or could be chosen as a result of a variety of policy decision‐making procedures. But I will make a stronger case; we have some evidence that carbon pricing would be adopted by groups that were deliberating in a procedurally just, democratic way. A decision is procedurally just when it appropriately treats the people who are affected by the decision, e.g. by allowing them input into the decision‐making process.^[^ ^29^ ^]^ This evidence comes from the Irish Citizens’ Assembly of 2016.^[^ ^30^ ^]^

Many campaigning groups are advocating for greater direct or deliberative democratic fora on climate issues. For instance, one of the activist group Extinction Rebellion's key demands is to create a Citizens’ Assembly on climate and ecological justice in the United Kingdom.^[^ ^31^ ^]^ Members of the umbrella group Rapid Transition Alliance (representing over a hundred international activist groups) have also advocated for a Citizens’ Assembly.^[^ ^32^ ^]^ In Canada, the activist Leap Manifesto^[^ ^33^ ^]^ calls for “town hall meetings” where local communities can determine how a transition would affect their future. Similar examples can be found worldwide. In contexts where campaigners advocate for greater (general) citizen say as opposed to a narrow, selected group (as with Citizens’ Assemblies), I actually think that this might not serve the goals of racial and climate justice campaigners for two reasons. First, greater citizen say in the United States has led to more veto points and local opposition to building, which slows or stops building that might be valuable, such as is the case with green infrastructure.^[^ ^55^ ^]^ Second, there is a moral argument, which is that if there is greater say among residents about what gets built, the opportunity to object will be disproportionately taken up by those with political and economic power, namely, the elite (and elderly) who have the time and resources to make their voices heard (Feldman and Turner^[^ ^52^ ^]^ consider this concern but ultimately dismiss it; I think their dismissal is too quick). In other words, a more general ability to voice objections in many cases could be expected to disproportionately increase power for those who already have it since those who self‐select into making their voices heard are unlikely to be representative. Note that this does not apply to Citizens’ Assemblies because the distribution of members is made to be (roughly) representative of the population along the lines of socioeconomic characteristics.

There is a good philosophical justification for policy‐evaluating procedures like Citizens’ Assemblies, a justification that follows what moral philosophers call “ideal observer theory”. The basic idea is that what we should do is not (necessarily) what we think we should do now or with our current information—what we should do is what we *would* want to do if we were apprised of the relevant facts and mechanisms relevant to our situation. The analogy is with an imaginary or hypothetical ideal observer who had your values or goals but knew more (or everything!) about the context of your action; what *she* would prescribe for you is what *you* should do. For instance, I might really want to eat some more lemon sorbet, but if I truly understood the impacts of eating it, I would not want to do it. Similarly, my ideal observer would not recommend that I keep eating that sweet, sweet sorbet. In the political realm, the question is not what we currently think is good policy, since few of us know what the current policies in any area *are*, let alone understand what they *should* be.

In a Citizens’ Assembly, a group of randomly selected, but socially stratified (i.e., trying to match population distribution in terms of age, gender, religion, ethnic background, citizenship status, etc.) members are brought together and paid for their time to answer some specific policy question such as “Which voting system is better?” Not every question is appropriate for such an assembly—“What should we do to make society better?” is too broad while “How should we design a power plant?” is too technical. These members then can ask various experts to give information (not policy suggestions or evaluations) that might be relevant. Sometimes, there is a set of experts chosen but, sometimes, the members are given options among some set of experts; in the best cases, the members can also request the experts that they want. The members go through several iterated processes of listening, deliberating, arguing, and voting on a range of issues relevant to their policy question.

Why is this morally relevant? Although most of us do not have the time to learn the details of various policy options, a Citizens’ Assembly can give us good evidence of which options, if we *were* to deliberate together, we might end up endorsing. Like an ideal observer, a Citizens’ Assembly is a well‐informed and well‐argued group that has (roughly) representative characteristics of the society from which they come. The key point is that the members exchange ideas and arguments, not just positions. This kind of working together is very different than simply polling various positions, where citizens are asked what their opinions on various policy options are even when these opinions are neither informed nor carefully thought through. If the ideal observer theory, or some theory like it, is true, then what we *should* do or endorse is what an informed body like a Citizens’ Assembly *would* do or endorse.

One could object that the results of a Citizens’ Assembly are not (or not sufficiently) procedurally just because a procedurally just process should include representatives of all affected groups—and the most important affected groups are future generations who have not yet been born. They will face the full brunt of climate impacts, so any process that excludes them, one might think, is illegitimate or procedurally insufficient.

I will provide both a more concessive and a more combative answer to this objection. First, the concessive answer is, yes, it would be more convincing or more democratically legitimate to have a procedure that includes all affected. On the one hand, no process fully includes everyone who is affected (we simply do not have the time and resources to weigh in on all policies that affect us, nor is it feasible for politicians to consult everyone even if citizens were to b interested in doing so). Indeed, even in the Irish Citizens’ Assemblies instance, it is not necessarily the case that all citizens are equally likely to participate.^[^ ^34^ ^]^ On the other hand, the objector might think that this is especially egregious in this instance because the preferences of future people may be very different from the preferences of current people. Of course, it is not possible for future or non‐existent people to participate in such procedures or for us to ask them what their preferences are or will be. One potential mechanism is to have some member(s) of a Citizens’ Assembly represent future people. Some parliaments have recently begun experimenting with representatives for future people, such as Wales, Hungary, and New Zealand.^[^ ^35^ ^]^ The concessive answer is that this is only a limited response to the democratic concern.

Second, the more combative answer is that, if done properly, we can include people who are good proxies for future generations on both scientific and theoretical grounds. These people are young people. On scientific grounds, young people can be expected to have similar attitudes to future generations because they stand to benefit greatly from mitigating climate change. This is because mitigation could move us away from IPCC scenarios with likely outcomes of 4.0°C, and such temperature rises have been said to be compatible with climate collapse.^[^ ^36^ ^]^ This means currently existing young people have the motivation to act on behalf of future generations. If they are properly informed through the Citizens’ Assembly process, they would also have the relevant epistemic similarity to future generations, thus jointly satisfying what Byskov and Hyams^[^ ^37^ ^]^ call a “hypothetical acceptance criterion” for future people. (It is worth noting that Byskov and Hyams^[^ ^37^ ^]^ come to a different conclusion; they believe that this shows communities already affected by climate change satisfy their criteria. I believe that this is true, but in light of current scientific literature, we can say that young people *globally* do.) Due to both the current scientific basis that climate change threatens young people in ways that align their motivations to future people and their capacity to be informed in ways that align their understanding to future people, they can reasonably represent future people in such processes.

So not only do some influential campaigning groups advocate for the formation of such groups, but there is also a good moral reason to take these groups seriously. And we have an example of such a national Citizens’ Assembly specifically in the context of climate policy.

This example is the Irish Citizens’ Assembly (100 members), which met in 2017 to discuss how Ireland might become an international leader in climate action and generated stunning and overwhelming agreement on a variety of measures. Most pertinent to the current topic, though, is that 89% of the members “recommended that there should be a tax on greenhouse gas emissions from agriculture [with] resulting revenue […] reinvested to support climate friendly agricultural practices” and 80% of members voting that they (themselves) “would be willing to pay higher taxes on carbon intensive activities”.^[^ ^30^ ^]^ That is much higher than most surveys done for carbon pricing, a difference I would attribute to the care, time, and information allowed for deliberation in this context. Obviously, not every country would vote as an Irish assembly would, and even within Ireland, we do not know whether a repeat assembly held on the same question with different participants would generate the same answers. This is just one instance of something that I believe is procedurally just; it is possible that, if re‐run at different times in different locales, different support from carbon pricing would occur. However, Citizens’ Assemblies are expensive, and the methodology is convincing, so we should not take this deliberative democratic exercise lightly, even if it is a lone case—especially because it was well‐run.

What can we conclude from this? Not only is carbon pricing *in principle* compatible with more direct or more deliberative democratic procedures (or, indeed, *less* direct or *less* deliberative democratic procedures), but we have evidence that it is *in fact* supported by such activities. These procedures are also philosophically justifiable using the moral framework of the ideal observer theory. The supermajoritarian support for carbon pricing in the Irish Citizens’ Assembly is evidence that, when regular citizens are apprised of relevant information and able to devote time to thinking through and discussing related issues, carbon pricing is a policy that many would come to support. Those of us who have spent less time understanding and debating the policy mechanisms should consider the possibility that we would also come to endorse such policies in similar circumstances.

## 3 Fundamental Change Takes Time

Having argued that carbon pricing is neither distributively nor procedurally unjust, it is worth considering what a carbon pricing critic might say. Such a critic might argue that, even if it were granted that carbon pricing did not set back distributive and other dimensions of justice, not setting back justice is aiming too low. Instead of just considering how we can incrementally become more just and merely avoiding unjust policies (call this “justice‐constrained” policy choice, with thanks to Ross Mittiga for the suggested terminology), we should aim for justice‐maximizing policy frameworks (call this “justice‐determinative” policy choice). I will respond that such criticism is reasonable in some policy domains, but it is not appropriate when discussing climate policy, due to the immediacy of this issue.

The idea is that this critic might object that what we have done so far is just check whether carbon pricing sets back the progress of certain kinds of justice. This kind of justice‐constrained policy choice reflects slogans like “no justice, no peace”, or the idea that if a policy is not (sufficiently) just, it should be taken off the table. But this would be insufficiently ambitious for this critic. Perhaps she would endorse a view like Naomi Klein's that we need nothing short of a framework that is a comprehensive “all‐out war” on a variety of injustices.^[^ ^4^ ^]^ Perhaps she would point out that a broader tent of activists might be more politically coherent and powerful. The kind of justice‐determinative policy choice she would advocate is high risk, high reward, where the only acceptable policy frameworks are those that increase the likelihood of reaching optimally overall just outcomes. This strategy reflects slogans like “system change, not climate change”, where the goal is to endorse frameworks that move societies toward outcomes that reflect a variety of forms of justice (e.g., racial justice, economic justice).

I believe that many campaigners are motivated by this kind of maximalist vision of justice. Indeed, I would grant that it might be instrumentally valuable in a policy ecosystem to have justice‐maximizing positions in discussion in order to expand the Overton window. It is certainly the case that a technocratic policy instrument like carbon pricing might seem like an unsatisfying or visionless alternative in this context (even if only as part of a policy portfolio). However, I think a more substantive response can be offered.

That response is that, while this kind of maximalist approach might be appropriate for domains or challenges for which there is time to coordinate and iterate policy frameworks, this is not the case with climate change. (I say “might be appropriate” because I am agnostic about whether this is true in general; what I do believe is that, in the current case, the downsides of a maximalist approach are decisive.) After all, both climate campaigners and climate experts agree that climate change requires urgent action (terms like “climate crisis” and “climate emergency” express this urgency for activists whereas scientists such as those behind the special report on 1.5 degrees tell us that climate models suggest we need to be rapidly decelerating emissions by 2030 with an aim to be at approximately net zero by 2050 to be compatible with 1.5 degrees).^[^ ^38^, ^39^, ^40^, ^41^ ^]^ We also know that climate outcomes have the potential to be disastrous^[^ ^36^ ^]^—indeed, they are already causing massive harms that are disproportionately borne by the global South (the recent 2022 flooding in Bangladesh being especially catastrophic). And we know that delayed action is costly.^[^ ^42^ ^]^ So, climate policy is one of the few policy domains where we are at a hinge point; delayed action is, to a first approximation, inefficacious action.

This sets the stage for an objection I call “(fundamental) change takes time”.^[^ ^8^ ^]^ The objection is that, in the context of climate change, trying to make fundamental changes to society requires time both (a) to agree on which changes to make and how and (b) even once that agreement is made, to iterate and refine the implementation of those changes. I conclude that, at least in the climate context, these frameworks advocating overall just outcomes would be too slow—or at least slower than various incremental policies *without* such grand overarching goals.

Consider the two points in turn. First, a more fundamental and comprehensive social change involves more decision points; thus, it allows more space for disagreement among advocates. Building consensus on a range of contested issues is intuitively slower than reaching an agreement on a single narrower issue, even among those who are on the “same side” or have the “same opponents”. Wolff,^[^ ^43^ ^]^ for instance, notes that it can be difficult when “coalitions set out broad agendas” which can easily lead to “distract[tions] by emerging crises, and numerous side issues”. This is easy to overlook when a group is united against the status quo, whether on narrow (polluted neighborhoods) or broad (capitalism) grounds. It is much easier to unite in opposition than to unite in favor, and it is easier to converge in favor of something narrow and simple than something broad and complex. This difficulty takes time, and in the context of climate change, I would suggest that we do not have the time for complex policy frameworks that are aimed at addressing many social ills. Note that I am not claiming that carbon pricing alone addresses climate change; this issue may well require a portfolio of climate policies working in conjunction, each contributing marginally to incentivizing green action and some reinforcing others. However, the point is that the goals of reducing emissions and adapting to climate impacts are complicated enough without requiring that policies simultaneously address other social ills. (I differ here from Boyce et al.^[^ ^16^ ^]^ in that I see some political costs to carving out particular benefits for racially or socioeconomically vulnerable groups. In particular, those kinds of carve‐outs might be objectionable to other groups and considered to reflect favoritism or special pleading. I would prefer the benefits to accrue to these vulnerable groups without explicit dispensation, and I believe that neutral policies would actually be disproportionately beneficial to vulnerable groups in effect, even if not with explicit intent.)

Indeed, economists point out that, in general, with multiple policy goals, one requires multiple policy instruments. They call this the “Tinbergen Rule”.^[^ ^44^ ^]^ Otherwise, one will be unable to determine which instrument to use since policy instruments are usually designed to achieve some particular kind of goal and rarely can optimize for multiple things at the same time. It is highly uncertain that a single policy instrument could meet the diverse goals that the maximalist is concerned with. A maximalist might answer that the proper response is a *variety* of policy instruments, but then a justice‐constrainer could say that (a) that is fully compatible with carbon prices being a component of a suite of policy instruments and (b) this acknowledges that the work required to implement the suite could reasonably be many times as much as that required to implement a single policy instrument.

Second, even if a campaign group manages to coordinate a particular framework that addresses a variety of social ills, more time is needed to refine and iterate it. By this, I mean two things. First, they need to make the case to the public that this is a valuable framework, which might take longer if it is a more complex and ambitious framework. Second, and this is crucial, if they have public support and buy‐in, they also need to implement it and more complex policies are less likely to succeed or be implementable immediately. Carbon prices would affect some sectors of the economy significantly. However, more broad plans that are also trying to address racial and gender justice or that are trying to limit capitalism are likely to require even more tinkering. There are, for instance, all kinds of ways that social changes can go wrong, all kinds of edge cases that might become salient that would not have been recognized beforehand, and all kinds of conflicts that could arise. All of these various considerations would likely take time to recognize, diagnose, and respond to.

Since a framework that tries to address a variety of social ills will (try to) change many social facts, there are more of these issues that might arise, leading to a process of adjustment and iteration. That is not straightforwardly a bad thing; we should want our policies to be sensitive to their implementation and to be adjusted on the basis of tensions that arise. However, this process is likely to slow the implementation of the framework and generate various kinds of objections and backlash.

In short, when an ambitious framework is proposed, we should expect that it is slower to develop and slower to enact than narrow (simpler) policies. While such frameworks may do more overall good (once they are developed and enacted), that is a trade‐off that should be taken seriously. In the context of climate change, this is especially worrying since action to address climate change must be soon if it is to be effective. This is why I call this the “(fundamental) change takes time” objection.

## 4 Conclusion

One of the intuitions that leads some to consider carbon pricing to be unjust is that they think carbon pricing is unfair. But I would suggest the opposite. What is unfair is letting people pollute for free. It's unfair partially because society has to pay instead,^[^ ^45^ ^]^ but it's also unfair because the people who generate the most emissions are already disproportionately wealthy.^[^ ^23^, ^46^ ^]^

So, in this context, what I have argued in this perspective—that carbon pricing is not unjust—is less surprising. Both in distributive and procedural justice terms, carbon pricing can be justified. In distributive justice terms, the revenues from a carbon price can be used to avoid net regressive effects. In procedural justice terms, I argued that the outcomes of the Irish Citizens’ Assembly suggest that many of us, if suitably informed, would support carbon pricing policies. When combined with an influential moral theory (ideal observer theory), this supports the claim that our actual (uninformed) selves should also support carbon pricing policies.

These justifications are obviously not enough to say that carbon pricing is always the best policy. However, it is enough to say that it has more strengths than are commonly recognized, that it should not be dismissed and that it has a legitimate spot within the portfolio of climate policy responses. My hope is that the kind of carbon pricing policy operational in places like British Columbia could be exported to other jurisdictions, but for the purposes of this perspective my goals were simply to demonstrate that justice as a consideration should not be an impediment to spreading such policies.

## Conflict of Interest

The author declares no conflict of interest.
